# Dr. Edwin Amsden

**Published:** 1910-09

**Authors:** 


Dr. Edwin Amsden, of San Diego, California, formerly of Allegan, Mich., died at the former place from senile debility, July 13, 1910, aged 84 years. He was a native of Gainesville, N. Y., the son of Elihu Amsden, the latter being a practitioner of prominence in that region during the earlier days. Dr. Edwin Amsden graduated in medicine at the University of Buffalo in 1853, and served first as assistant surgeon, then as surgeon, of the 136th New York regiment of volunteer infantry, during the Civil War. After his return to civil life, he located at Allegan, where he enjoyed a large practice for many years. Later in life he retired and sought the milder climate of southern California in which to spend the remainder of his days.

